# Efficacy and safety of oral sildenafil in children with Down syndrome and pulmonary hypertension

**DOI:** 10.1186/s12872-017-0569-3

**Published:** 2017-07-04

**Authors:** Maurice Beghetti, Andrzej Rudzinski, Min Zhang

**Affiliations:** 1 Pediatric Cardiology Unit, Department of Child and Adolescent, University of Geneva, Geneva, Switzerland; 2 Pediatric Cardiology, Jagiellonian University, Gołębia 24, 31-007 Cracow, Poland; 3 Pfizer Inc, 10646 Science Center Dr, La Jolla, San Diego, CA 92121 USA

**Keywords:** Sildenafil, Down syndrome, Pulmonary hypertension, Children

## Abstract

**Background:**

Despite the increased risk for pulmonary hypertension in children with Down syndrome, the response to treatment with targeted therapies for pulmonary hypertension in these patients is not well characterized. The **S**ildenafil in **T**reatment-naive children, **A**ged 1–17 years, with pulmona**r**y ar**t**erial hyperten**s**ion (STARTS-1) trial was a dose-ranging study of the short-term efficacy and safety of oral sildenafil in children with pulmonary arterial hypertension. We assessed the safety and efficacy of oral sildenafil in children with Down syndrome and pulmona**r**y ar**t**erial hyperten**s**ion.

**Methods:**

This was a post-hoc analysis of children with Down syndrome and pulmonary arterial hypertension enrolled in the STARTS-1 trial. Mean pulmonary arterial pressure (mPAP), pulmonary vascular resistance index (PVRI), and cardiac index (CI) were assessed at baseline and following 16 weeks of treatment with sildenafil.

**Results:**

Of 234 patients randomized and treated in the STARTS-1 trial, 48 (20.5%) had Down syndrome. Although sildenafil produced dose-related reductions in PVRI and mPAP, compared with placebo, in non–Down syndrome patients and children developmentally able to exercise, this was not satisfactorily marked in patients with Down syndrome. The dose-related reductions in PVRI, compared with placebo, occurred in all subgroups, with the exception of the Down syndrome subgroup. Sildenafil appeared to be well tolerated in the Down syndrome subpopulation and the most frequently reported AEs were similar to those reported for the entire STARTS-1 population.

**Conclusion:**

Sildenafil treatment for 16 weeks had no effect on PVRI or mPAP in children with Down syndrome and pulmonary arterial hypertension. The results suggest that children with Down syndrome may be less responsive to sildenafil for pulmonary arterial hypertension, but the incomplete work-up for the etiology of pulmonary arterial hypertension may have introduced a potential bias.

**Trial registration:**

Study received, September 8, 2005 (retrospectively registered); Study start, August 2003; ClinicalTrials.gov identifier, NCT00159913.

**Electronic supplementary material:**

The online version of this article (doi:10.1186/s12872-017-0569-3) contains supplementary material, which is available to authorized users.

## Background

Down syndrome (DS) is the most commonly occurring chromosomal abnormality at birth, with about 1 in 700 babies born each year in the United States having DS [1]. In children with DS, the frequency of congenital heart defects, mainly atrioventricular septal and ventricular septal defects, is high [2], and many children with DS have a high prevalence of associated respiratory problems, in particular, upper airway obstruction [3]. Findings from a recent study of patients with DS living in Mexico City (high altitude) showed that 40% had congenital heart disease and 80% had pulmonary hypertension (PH) assessed by echocardiography [4]. Children with DS have a higher risk for developing PH due, in part, to these congenital heart defects [5]. Other factors that may play a role in the higher risk for PH in children with DS include upper airway obstruction, lung hypoplasia, gastroesophageal reflux, abnormal pulmonary vascular function, obstructive sleep apnea (OSA) [6–8], and an imbalance in mediators, such as nitric oxide, thromboxane A2, and prostacyclin [9–11], which have been implicated in the development of PH [12]. Despite the increased risk for PH in children with DS, the response to treatment with targeted therapies for PH in these patients is not well characterized. The **S**ildenafil in **T**reatment-naive children, **A**ged 1–17 years, with pulmona**r**y ar**t**erial hyperten**s**ion (STARTS-1) trial was a dose-ranging study of the short-term efficacy and safety of oral sildenafil in pediatric patients with pulmonary arterial hypertension (PAH) [13]. The purpose of this report is to present the results of a post-hoc analysis of the patients with DS enrolled in STARTS-1.

## Methods

The STARTS-1 study design has been previously published [13]. Briefly, children aged 1 to 17 years with idiopathic PAH (IPAH), heritable PAH (HPAH), or PAH associated with congenital heart defects (PAH-CHD) were included. To be included, children had to have a mean pulmonary arterial pressure (mPAP) ≥25 mmHg at rest, pulmonary capillary wedge pressure ≤ 15 mmHg, and pulmonary vascular resistance index (PVRI) ≥3 Wood units × m^2^. Children who were developmentally able to exercise had to have a peak venous oxygen saturation (pVO_2_) ≥10 mL/kg/min and ≤20 mL/kg/min during a screening cardiopulmonary exercise test (CPET). Children were randomized to placebo or sildenafil low, medium, and high doses that were selected to achieve maximum plasma concentrations of 47, 140, and 373 ng/mL, respectively, at steady state. Patients with body weight between 8 and 20 kg were randomly assigned to receive medium- (10 mg TID) or high -dose (20 mg TID) sildenafil; patients between 20 and 45 kg were randomly assigned to receive low- (10 mg TID), medium- (20 mg TID), or high -dose (40 mg TID) sildenafil; and patients >45 kg were randomly assigned to receive low- (10 mg TID), medium- (40 mg TID), or high -dose (80 mg TID) sildenafil.

Randomization was stratified by weight and developmental ability to perform a CPET (assessed using bicycle ergometry). The primary outcome measure of pVO_2_ was assessed by CPET only in children who were able to exercise reliably at baseline and at week 16. This group was termed “developmentally able”. The group termed “non-developmentally able” included children who were too young or who had other concomitant conditions such as DS, which prevented them from performing the CPET test as judged by the investigator. In all patients, secondary outcome measures of mPAP, PVRI, and cardiac index (CI) were assessed at baseline and at week 16. Complete physical examinations and laboratory tests were performed in all patients, and investigators recorded adverse events (AEs) and serious AEs (SAEs) throughout the study.

The secondary outcome measures were evaluated by using analysis of covariance (ANCOVA) in which treatment comparisons were made between each of the sildenafil groups (low, medium, and high dose) and the placebo group, and between the combined sildenafil group and the placebo group. For PVRI and CI, changes from baseline at week 16 were log-transformed before data analysis due to normality assumptions that were not met for untransformed data. For non-DS subjects, ANCOVA was conducted with independent variables of etiology, weight group, capability of performing the CPET, log-transformed baseline value, and treatment; for DS subjects, due to the limited number of subjects, ANCOVA was conducted with independent variables of log-transformed baseline value and treatment. For mPAP, for non-DS subjects, ANCOVA was conducted with independent variables of etiology, weight group, capability of performing the CPET, and treatment; for DS subjects, ANCOVA was conducted with the independent variables of etiology and treatment. Statistical hypothesis testing was performed at the 2-side 5% significance level. Descriptive statistics were used to describe AEs and SAEs.

## Results

Of 234 patients randomized and treated in the STARTS-1 trial, 48 (20.5%) had DS. Of the patients with DS, 15 were aged <7 years and 33 were aged ≥7 years; only 2 of the 48 patients were developmentally able to exercise (1 patient each in sildenafil low- and medium-dose groups). The number of patients with DS diagnosed with IPAH/HPAH, PAH-CHD with surgical repair, and PAH-CHD with congenital systemic-to-pulmonary shunt was 5 (10.4%), 16 (33.3%), and 27 (56.3%), respectively. Approximately 60% of the non-DS patients had non-IPAH/HPAH, whereas approximately 90% of the DS patients had non-IPAH/HPAH. Therefore, there was an imbalance across the etiology groups. Baseline demographic and clinical characteristics of the DS and non-DS patients were similar **(**Tables 1 and 2 **)**.

**Table 1:** Baseline demographics

	Non–Down Syndrome (*n* = 186)	Down Syndrome (*n* = 48)
Age, y, n (%)		
1–4	26 (14)	9 (19)
5–12	101 (54)	25 (52)
13–17	59 (32)	14 (29)
Mean (SD)	9.8 (4.3)	8.8 (4.5)
Range	1–17	1–16
Race, n (%)		
White	65 (35)	32 (67)
Black	4 (2)	1 (2)
Asian	38 (20)	3 (6)
Other	79 (43)	12 (25)
BMI, mean (SD)	16.6 (3.7)	19.0 (4.1)
Range	10.6–36.8	13.3–30.0
Etiology		
Idiopathic/heritable	73 (39)	5 (10)
Associated with CHD		
Surgical repair	51 (27)	16 (33)
Unrepaired	62 (33)	27 (56)

*BMI* body mass index, *CHD* congenital heart disease, *SD* standard deviation

**Table 2:** Baseline clinical characteristics

	Non–Down Syndrome		Down Syndrome	
Parameter	n	Mean (SD)	n	Mean (SD)
mPAP, mm Hg	184	61.5 (23.0)	47	63.6 (16.7)
mSAP, mm Hg	184	75.3 (14.3)	47	67.9 (12.5)
RAP, mm Hg	184	8.3 (5.1)	47	7.5 (2.8)
mPAP/mSAP	184	0.8 (0.3)	47	0.9 (0.2)
PCWP, mm Hg	183	9.8 (3.8)	47	9.4 (2.9)
CI, L/min/m^2^	181	3.4 (1.5)	45	3.6 (2.3)
PVRI, dyn∙s/cm^5^/m^2^	177	1441.3 (1052.7)	45	1638.4 (1273.2)
SVRI, dyn∙s/cm^5^/m^2^	181	1822.4 (860.1)	45	1669.0 (852.6)
PVRI/SVRI	176	0.8 (0.7)	45	1.2 (1.3)
sPAP/sBP	184	0.9 (0.3)	47	0.9 (0.2)
dPAP/dBP	184	0.7 (0.3)	47	0.8 (0.2)
Peak VO_2_, mL/kg/min	113	18.4 (4.2)	2	12.4 (0.9)
Time to maximum peak VO_2_	113	466.7 (133.5)	2	445.0 (183.9)
% Predicted peak VO_2_	113	46.7 (11.4)	2	36.0 (5.1)
WHO Functional Class, n (%)	*N* = 183		*N* = 48	
Class I	57 (31)		18 (38)	
Class II	95 (52)		25 (52)	
Class III	30 (16)		5 (10)	
Class IV	1 (1)		0	

*CI* cardiac index, *dBP* diastolic blood pressure, *dPAP* diastolic pulmonary artery pressure, *mPAP* mean pulmonary artery pressure, *mSAP* mean systolic arterial pressure, *PCWP* pulmonary capillary wedge pressure, *PVRI* pulmonary vascular resistance index, *RAP* right arterial pressure, *sBP* systolic blood pressure, *sPAP* systolic pulmonary artery pressure, *SVRI* systemic vascular resistance index

**Fig. 1:**
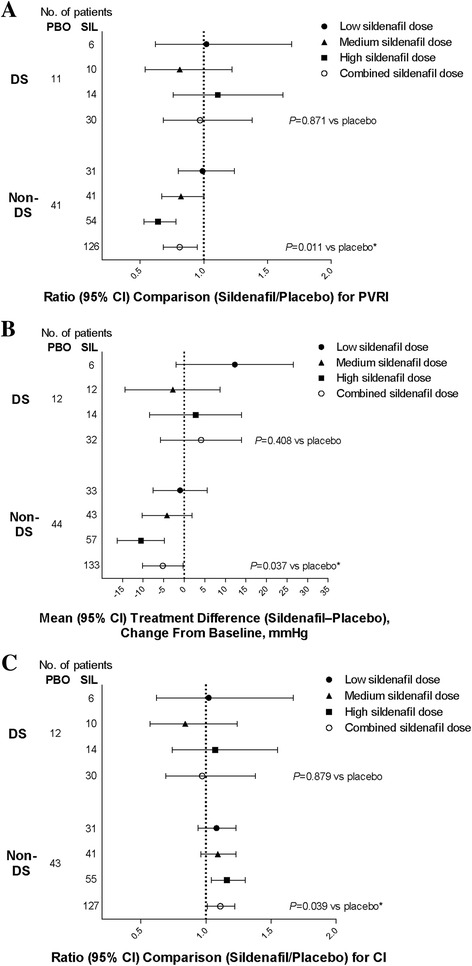
Changes from baseline at week 16 in **a**) PVRI, **b**) mPAP, and **c**) CI in DS versus non-DS patients. Treatment comparisons for pulmonary vascular resistance and cardiac index are in the form of ratios as the analyses were conducted on log transformed data. A log transformation of the week-16 data was used to achieve a normal distribution of the data. Log baseline value was included as a covariate in the analysis. DS = Down syndrome; PBO = placebo; CI = cardiac index; mPAP = mean pulmonary artery pressure; PVRI = pulmonary vascular resistance index; SIL = sildenafil

Sildenafil produced a trend of dose-related reductions in PVRI and mPAP compared with placebo in developmentally able and non-DS patients but not in patients with DS **(**Table 3 and Fig. 1 **)**. Similarly, among non-IPAH/HPAH patients, sildenafil produced a trend in dose-related improvements in PVRI and mPAP compared with placebo in non-DS patients but not in patients with DS (Table 4). Further analysis by etiology of the hemodynamic endpoints was performed with the DS children excluded to assess if this subpopulation affected the observed treatment responses.

**Table 3:** Treatment comparisons to placebo for PVRI, mPAP, and CI in the Down syndrome and non–Down syndrome populations

		Comparison to Placebo (95% CI)			
			All Non–Down Syndrome	Non–Down Syndrome	
Parameter	Treatment Group	Down Syndrome		Developmentally Able	IPAH/HPAH
PVRI, ratio active/placebo	Low Dose	1.02 (0.62, 1.69) (*n* = 6)	0.99 (0.80, 1.24) (*n* = 31)	0.96 (0.74, 1.24) (*n* = 24)	0.96 (0.67, 1.38) (*n* = 11)
	Medium Dose	0.81 (0.54, 1.22) (*n* = 10)	0.82 (0.67, 1.00) (*n* = 41)	0.78 (0.61, 1.00) (*n* = 27)	0.75 (0.54, 1.03) (*n* = 17)
	High Dose	1.11 (0.76, 1.62) (*n* = 14)	0.64 (0.53, 0.78) (*n* = 54)	0.56 (0.43, 0.72) (*n* = 26)	0.67 (0.50, 0.90) (*n* = 23)
	Combined Dose	0.97 (0.68, 1.38) (*n* = 30)	0.81 (0.68, 0.95) (*n* = 126)	0.74 (0.60, 0.91) (*n* = 75)	0.78 (0.60, 1.02) (*n* = 51)
mPAP, difference from placebo, mmHg	Low Dose	12.3 (−2.0, 26.5) (*n* = 6)	−0.99 (−7.55, 5.56) (*n* = 33)	−2.4 (−10.1, 5.3) (*n* = 26)	−1.0 (−11.0, 8.9) (*n* = 12)
	Medium Dose	−2.8 (−14.4, 8.7) (*n* = 12)	−4.15 (−10.15, 1.85) (*n* = 43)	−7.7 (−15.3, −0.2) (*n* = 28)	−2.8 (−11.7, 6.1) (*n* = 18)
	High Dose	2.8 (−8.4,13.9) (*n* = 14)	−10.52 (−16.25, −4.79) (*n* = 57)	−17.2 (−24.9, −9.6) (*n* = 27)	−7.7 (−16.0, 0.7) (*n* = 24)
	Combined Dose	4.1 (−5.8, 13.9) (*n* = 32)	−5.22 (−10.12, −0.32) (*n* = 133)	−9.6 (−15.7, −3.5) (*n* = 79)	−3.8 (−11.2, 3.5) (*n* = 54)
CI, ratio active/placebo	Low Dose	1.02 (0.62, 1.67) (*n* = 6)	1.08 (0.94, 1.23) (*n* = 31)	1.10 (0.94, 1.27) (*n* = 24)	1.07 (0.87, 1.31) (*n* = 12)
	Medium Dose	0.84 (0.57, 1.24) (*n* = 10)	1.09 (0.96, 1.23) (*n* = 41)	1.09 (0.95, 1.27) (*n* = 27)	1.24 (1.02, 1.50) (*n* = 17)
	High Dose	1.07 (0.74, 1.55) (*n* = 14)	1.16 (1.04, 1.30) (*n* = 55)	1.25 (1.08, 1.45) (*n* = 27)	1.35 (1.13, 1.62) (*n* = 23)
	Combined Dose	0.97 (0.69, 1.38) (*n* = 30)	1.11 (1.01, 1.22) (*n* = 127)	1.14 (1.01, 1.29) (*n* = 76)	1.21 (1.03, 1.43) (*n* = 52)

*CI* cardiac index, *HPAH* heritable pulmonary arterial hypertension, *IPAH* idiopathic pulmonary arterial hypertension, *mPAP* mean pulmonary artery pressure, *PVRI* pulmonary vascular resistance index

**Table 4:** Treatment comparisons to placebo for PVRI, mPAP, and CI in the non-IPAH/HPAH Down syndrome and non-Down syndrome populations

Parameter	Treatment Group	Comparison to Placebo (95% CI)	
		Down Syndrome	All Non–Down Syndrome
PVRI, ratio active/placebo	Low Dose	1.01 (0.59, 1.71) (*n* = 6)	0.98 (0.74, 1.29) (*n* = 20)
	Medium Dose	0.80 (0.51, 1.27) (*n* = 9)	0.87 (0.67, 1.14) (*n* = 24)
	High Dose	1.08 (0.71, 1.64) (*n* = 13)	0.62 (0.49, 0.80) (*n* = 31)
	Combined Dose	0.95 (0.65, 1.41) (*n* = 28)	0.81 (0.65, 1.00) (*n* = 75)
mPAP, difference from placebo, mmHg	Low Dose	10.87 (−3.21, 24.94) (*n* = 6)	−1.90 (−10.48, 6.67) (*n* = 21)
	Medium Dose	−4.44 (−16.34, 7.47) (*n* = 11)	−4.82 (−13.01, 3.36) (*n* = 25)
	High Dose	0.48 (−10.98, 11.95) (*n* = 13)	−11.41 (−19.07, −3.75) (*n* = 33)
	Combined Dose	2.30 (−7.79, 12.40) (*n* = 30)	−6.04 (−12.68, 0.59) (*n* = 79)
CI, ratio active/placebo	Low Dose	1.01 (0.61, 1.70) (*n* = 6)	1.11 (0.94, 1.32) (*n* = 19)
	Medium Dose	0.82 (0.54, 1.24) (*n* = 9)	1.02 (0.87, 1.20) (*n* = 24)
	High Dose	1.07 (0.72, 1.58) (*n* = 13)	1.08 (0.93, 1.26) (*n* = 32)
	Combined Dose	0.96 (0.67, 1.39) (*n* = 28)	1.07 (0.94, 1.22) (*n* = 75)

*CI* cardiac index, *HPAH* heritable pulmonary arterial hypertension, *IPAH* idiopathic pulmonary arterial hypertension, *mPAP* mean pulmonary artery pressure, *PAH-CHD* pulmonary arterial hypertension associated with congenital heart defects, *PVRI* pulmonary vascular resistance index

The most frequently reported AEs were diarrhea, vomiting, bronchitis, laryngitis, nasopharyngitis, cough, and headache **(**Table 5 **)**. None appeared to be dose related and the majority of AEs were of mild or moderate intensity. Four patients with DS reported SAEs. Two SAEs were considered treatment related (both in the high-dose sildenafil group): stridor in 1 patient during the first week of therapy and ventricular arrhythmia in the other patient. The other 2 patients received placebo and the SAEs were considered unrelated to treatment.

**Table 5:** Adverse events by treatment group occurring in ≥2 patients with Down syndrome

		Sildenafil Dose		
Adverse Event, n (%)	Placebo (*n* = 12)	Low (*n* = 7)	Medium (*n* = 12)	High (*n* = 17)
Conjunctivitis	1 (8)	0	0	2 (12)
Diarrhea	2 (17)	1 (14)	1 (8)	4 (24)
Vomiting	0	1 (14)	1 (8)	3 (18)
Pharyngitis	0	1 (14)	2 (17)	0
Upper respiratory tract infection	1 (8)	2 (29)	3 (25)	0
Pneumonia, bacterial	2 (17)	0	0	0
Headache	1 (8)	1 (14)	1 (8)	2 (12)

Includes data up to 7 days after last dose of sildenafil

## Discussion

PAH is a chronic disorder characterized by a progressive increase in pulmonary vascular resistance leading to right heart failure and, if left untreated, death [12]. Children with DS are at a high risk for PH^2^ but the response to treatment with targeted therapies for PAH in pediatric DS patients is not well characterized. The STARTS-1 trial evaluated the effects of low, medium, and high oral doses of sildenafil in pediatric patients with PAH, including 48 patients with DS [13]. For the primary endpoint, percent change in pVO_2_ from baseline to week 16, assessed by CPET, the combined sildenafil doses displayed a 7.7% (95% CI: –0.19, 15.60) improvement over placebo, but failed to achieve statistical significance (*P* = 0.056). CPET was performed only in children old enough and able to exercise reliably. However, secondary endpoints, including mPAP and PVRI, were assessed in all enrolled patients and improved with medium and high doses of sildenafil treatment.

The present post-hoc analysis compared the effects of sildenafil in the DS cohort with other subpopulations enrolled in the STARTS-1 trial. Exercise capacity is difficult to measure in patients with DS. Intellectual disability and failure to understand the test procedure can have major effects on the results of exercise tests [14]. Because there were only 2 patients with DS that were developmentally able to exercise, changes in pVO_2_ were not analyzed. For the secondary endpoints of PVRI and mPAP, sildenafil produced similar dose-dependent reductions compared with placebo for all subgroups except the DS subgroup. Similar to the results of the STARTS-1 trial, improvements in these hemodynamic parameters in the non-DS subgroups were only seen with the medium and high doses of sildenafil. The overall lack of effect of sildenafil in children with DS suggests that the response to PAH therapy may be different in this group of patients compared with non-DS children.

The effectiveness of PAH therapies in patients with DS have been mixed and often include adult DS patients. In an open-label, observational study, endothelin receptor antagonism with bosentan or sitaxentan improved 6-min walk distance (6MWD) and WHO functional class in adult patients with PAH-CHD with or without DS [15]. In another open-label study of 24 adult patients with DS and Eisenmenger syndrome, bosentan therapy resulted in increased 6MWD during the first 3 months of bosentan treatment but slowly returned to baseline after 26 and 52 weeks of therapy [16]. Additionally, findings from an observational study of 7 adults with DS and Eisenmenger syndrome demonstrated improved exercise capacity and oxygen saturation after exercise with long-term bosentan therapy [17]. Duffels et al. [18] retrospectively assessed changes from baseline in 6MWD following 6 months of treatment with bosentan in 58 adult patients with PAH due to CHD, 28 patients with DS, and 30 patients without DS. 6MWD improved significantly from baseline at 6 months in the non-DS patients but did not change in the DS patients. In addition, Eipe et al. [19] reported a recent case of severe PAH in an 11-year-old girl with DS who continued to worsen despite maximal PAH therapy with warfarin, sildenafil, bosentan, and inhaled epoprostenol. The patient also had a patent foramen ovale and was diagnosed with mild OSA possibly due to enlarged tonsils. Following adenotonsillectomy and initiation of continuous positive airways pressure to treat the OSA, her PAH stabilized.

In the STARTS-1 trial, there was no prespecified requirement to exclude OSA and/or upper airway obstruction that is not obviously symptomatic in DS. Thus, it is unknown if some of the DS patients in the study may have some type of upper airway obstruction that could explain the lack of sildenafil response. Hawkins et al. [3] reported in his series that 18 of 25 DS patients screened for PH had some sort of airway disease, including 12 who had upper airway obstruction. Even mild upper airway obstruction may play a role in the development of PH in this population of DS patients, particularly if associated with CHD.

OSA has a higher prevalence in children with DS (45% to 63%) [6, 20, 21] than in otherwise healthy children (2%) [22], which may be related to mandibular hypoplasia, a large tongue with a relatively small upper airway and generalized muscular hypotonia, resulting in collapse of the airway during inspiration [21, 23]. If snoring is not present, OSA is at times clinically not suspected; however, this may contribute to unexplained PH [21]. Large tonsils and adenoids appear to precipitate OSA and upper airway obstruction in children that may lead to PH.

There are limited data regarding the effects of PAH therapies in patients with OSA or upper airway obstruction. Roizenblatt et al. [24] assessed the effect of sildenafil 50 mg at bedtime on nocturnal respiratory and desaturation events in 14 middle-aged men with severe OSA in a double-blind, placebo-controlled, crossover study. Sildenafil significantly increased the frequency and duration of these events, which the authors speculated may be due to increased nasal congestion. The nasal congestion may even augment upper airway obstruction and indeed may be deleterious for these patients. These problems may, in part, explain the limited effect of sildenafil in this population.

The results of the present analysis suggest that children with DS and PAH may be less responsive to sildenafil therapy. This is also consistent with previous studies that have documented reduced endothelium-dependent vasodilation in DS patients compared with age-matched healthy individuals [25] and an imbalance in the synthesis of the vasoconstrictor thromboxane A2 compared with the vasodilator prostacyclin in children with DS [10]. In addition, children with PAH-CHD and DS are less responsive to inhaled nitric oxide than children with PAH-CHD but without DS [26]. Finally, levels of circulating endothelial progenitor cells, which may play a role in endothelial repair and be predictive of cardiovascular events and death from cardiovascular causes [27] are low and more susceptible to oxidative damage in individuals with DS compared with healthy individuals [28]. Moreover, circulating endothelial progenitor cells are low in adult patients with Eisenmenger syndrome compared with healthy individuals, and those with DS had even fewer endothelial progenitor cells [29]. Hence, physicians need to carefully monitor vasodilator therapy effectiveness when treating patients with DS and PAH.

One potential reason for the increased incidence of PAH in children with DS is that children with DS may have pulmonary hypoplasia [8, 11]. One study demonstrated that respiratory architecture develops poorly in infants with DS [8]. Pulmonary hypoplasia can lead to PAH because of enlarged alveoli and a decrease in the number of alveoli, which means that capillaries cover a proportionally smaller surface area of alveoli [11]. However, other pathologies presenting lung hypoplasia, such as congenital diaphragmatic hernia, have shown response to sildenafil suggesting that the latter may be not the only reason for non-response in DS [30]. Additional studies are warranted to determine whether pulmonary hypoplasia plays a role in the limited effect of sildenafil in DS children.

Sildenafil appeared to be well tolerated in this subpopulation of patients with DS. The most frequently reported AEs in the DS cohort were similar to those reported for the entire STARTS-1 population [13]. Most AEs were of mild or moderate intensity.

Limitations of this study include a small sample size and the inability to analyze the primary efficacy outcome of pVO_2_ in the DS cohort. Another potential limitation is the difference in the baseline characteristics between the DS and non-DS groups. An imbalance in etiology was observed with approximately 60% of the non-DS patients having non-IPAH/HPAH, whereas approximately 90% of the DS patients having non-IPAH/HPAH. The STARTS-1 study inclusion criteria did not specifically ask for a complete evaluation of DS and its pulmonary abnormalities. OSA and upper airway obstruction were not completely ruled out in DS patients from the STARTS-1 study; these lung problems may have contributed to the lack of sildenafil response. Recent studies also have identified histologic evidence of alveolar simplification, prominent bronchial vessels, intrapulmonary bronchopulmonary anastomoses, and impaired lung vascular growth and signaling, which may increase the risk of PAH and potentially explain limited effect of sildenafil [31, 32]. In addition, due to the inherent limitations of post-hoc analyses, the results of this study should be regarded as hypothesis generating.

## Conclusions

Sildenafil monotherapy treatment for 16 weeks had no significant effect on PVRI or mPAP in children with DS and PAH. Further studies are required to address and confirm these results and better evaluate whether dosing and/or respiratory screenings to exclude other potential etiologies of PH may affect sildenafil efficacy.

## Additional file

Additional file 1: List of investigators and corresponding ethics committees or institutional review boards. (PDF 123 kb)

## Abbreviations

6MWD: 6-min walk distance
ANCOVA: analysis of covariance
CI: cardiac index
CPET: cardiopulmonary exercise testing
DS: Down syndrome
HPAH: heritable PAH
IPAH: idiopathic PAH
mPAP: mean pulmonary arterial pressure
OSA: obstructive sleep apnea
PAH: pulmonary arterial hypertension
PAH-CHD: PAH associated with congenital heart defects
PH: pulmonary hypertension
pVO_2_: peak venous oxygen saturation
PVRI: pulmonary vascular resistance index
STARTS-1: **S**ildenafil in **T**reatment-naive children, **A**ged 1–17 years, with pulmona**r**y ar**t**erial hyperten**s**ion trial
