# Supplementary material for: Efficacy and safety of oral sildenafil in children with Down syndrome and pulmonary hypertension
Source: BMC Cardiovasc Disord. 2017 Jul 4;17:177. doi: 10.1186/s12872-017-0569-3 (PMC5496590; doi:10.1186/s12872-017-0569-3)
Supplement: Additional file 1: — List of investigators and corresponding ethics committees or institutional review boards. (PDF 123 kb) [file 12872_2017_569_MOESM1_ESM.pdf]

090177e1806e1c23\Approved\Approved On: 01-Dec-2008 01:38 (GMT)

# A4 LIST OF INVESTIGATORS AND CORRESPONDING ETHICS COMMITTEES OR INSTITUTIONAL REVIEW BOARDS

## Australia

**Coordinating Investigators:**
<None Entered>

| Center | Principal Investigator | Co-Investigator(s) | Sub-Investigator(s) | Address(es) | Institutional Review Board or Ethics Committee Address(es) |
|---|---|---|---|---|---|
| 1018 * | Prof. Daniel James Penny | | Dr. James Edward Elder<br>Natalie Lindsay<br>Michelle L. Rose<br>Dr. Robert Gideon Weintraub<br>Naomi B. Whittington<br>Ms. Sarah Wilson | The Royal Children's Hospital, Cardiology Department<br>Flemington Road<br>Parkville, VIC 3052<br>AUSTRALIA | Royal Children's Hospital<br>Royal Children's Hospital Ethics in Human Research Committee<br>Flemington Road<br>Parkville, VIC 3052<br>AUSTRALIA |

* Did not randomize subjects

090177e1806e1c23\Approved\Approved On: 01-Dec-2008 01:38 (GMT)

## Brazil

**Coordinating Investigators:**
<None Entered>

| Center | Principal Investigator | Co-Investigator(s) | Sub-Investigator(s) | Address(es) | Institutional Review Board or Ethics Committee Address(es) |
|---|---|---|---|---|---|
| 1058 | Dr. Maria Virgínia Tavares Santana | | Dr. Almir S. Ferraz<br>Ricardo F. Martins<br>Dr. Romeu S. Meneghelo<br>Dr. Carlos Augusto Cardoso Pedra<br>Dr. Lily E. M. Rabanal<br>Dr. Taciana C. C. Dona<br>Dr. Marly A. Miaira<br>Dr. Juliana A. Sabino | Instituto Dante Pazzanese de Cardiologia<br>Av Doutor Dante Pazzanese, 500 - Prédio I<br>Vila Mariana<br>São Paulo, SP 04012-909<br>BRAZIL | Comitê de Ética em Pesquisa do Instituto Dante Pazzanese de Cardiologia<br>Av. Doutor Dante Pazzanese, 500 - Prédio I<br>Vila Mariana<br>São Paulo, SP 04012-909<br>BRAZIL |

* Did not randomize subjects

## Canada

**Coordinating Investigators:**
<None Entered>

| Center | Principal Investigator | Co-Investigator(s) | Sub-Investigator(s) | Address(es) | Institutional Review Board or Ethics Committee Address(es) |
|---|---|---|---|---|---|
| 1032 * | Dr. Tilman Humpl | | Dr. Andrew Redington | The Hospital for Sick Children<br>555 University Avenue<br>Toronto, ON M5G 1X8<br>CANADA | The Hospital for Sick Children<br>Research Ethics Board<br>555 University Avenue<br>Toronto, ON M5G 1X8<br>CANADA |
| 1033 | Dr. James Y. Coe | | | University of Alberta, Walter Mackenzie Health Sciences Centre<br>Room 4C236<br>8440-112 Street<br>Edmonton, AB T6G 2B7<br>CANADA | Health Research Ethics Board<br>Biomedical Research, University of Alberta<br>Walter Mackenzie Health Science Centre<br>132 University Camous NW<br>Room 2J2.27<br>Edmonton, AB T6G 2R7<br>CANADA |
| 1035 * | Dr. George Sandor | | Martin C.K. Hosking MD | British Columbia Children's Hospital<br>Room 1A13<br>4480 Oak Street<br>Vancouver, BC V6H 3V4<br>CANADA | Children's and Women's Health Centre of BC Research Review Committee<br>Room 202<br>950 West 28th Avenue<br>Vancouver, British Columbia V5Z 4H4<br>CANADA<br><br>Clinical Research Ethics Board<br>#210-828 W. 10th Avenue<br>Vancouver, BC V5Z 1L8<br>CANADA |

* Did not randomize subjects



090177e1806e1c23\Approved\Approved On: 01-Dec-2008 01:38 (GMT)

090177e1806e1c23\Approved\Approved On: 01-Dec-2008 01:38 (GMT)

## Chile

**Coordinating Investigators:**
<None Entered>

| Center | Principal Investigator | Co-Investigator(s) | Sub-Investigator(s) | Address(es) | Institutional Review Board or Ethics Committee Address(es) |
|---|---|---|---|---|---|
| 1060 * | Leopoldo Romero | | Luis Cárdenas<br>Dr. Bernardita Lopetegui | Hospital Luis Calvo Mackenna<br>Servicio Cardiovascular<br>Antonio Varas 360<br>Providencia<br>Santiago, RM<br>CHILE | Comité Ético Científico Pediátrico<br>Antonio Varas 360<br>Santiago, Chile<br>CHILE |
| 1061 | Lida Toro | | Dr. Alex Alcantara<br>María Elisa Castillo<br>Francisco Garay<br>Luis Garay<br>Fernando Herrera<br>Pamela Zelada | Hospital Dr. Sótero del Río<br>Cardiología Pediátrica<br>Concha y Toro 3459<br>Puente Alto<br>Santiago, RM<br>CHILE | Comité de Evaluación Etico Científico<br>Hospital Dr. Sótero del Río Servicio de Salud Metropolitano Sur Oriente<br>Av. Concha y Toro 3459,<br>Paradero 30 Vicuña Mackenna<br>Puente Alto, Santiago<br>CHILE |

* Did not randomize subjects

090177e1806e1c23\Approved\Approved On: 01-Dec-2008 01:38 (GMT)

## Colombia

**Coordinating Investigators:**
<None Entered>

| Center | Principal Investigator | Co-Investigator(s) | Sub-Investigator(s) | Address(es) | Institutional Review Board or Ethics Committee Address(es) |
|---|---|---|---|---|---|
| 1055 | Dr. Luz Elena Arbelaez | | Dr. Alfonso Valencia | Hospital Santa Clara<br>Empresa Social del Estado<br>Carrera 15 No. 1-59 Sur<br>Bogota, Cundinamarca 0<br>COLOMBIA | Comite de Etica en Investigacion - Hospital Santa Clara - Empresa Social del Estado<br>Hospital Santa Clara<br>Empresa Social del Estado<br>Carrera 15 No. 1 - 59 Sur<br>Bogota, Cundinamarca<br>COLOMBIA |
| 1056 | Dr. Alberto Garcia | | Dr. Oscar Baron Puentes<br>Dr. Miguel Ronderos | Fundacion Cardio Infantil<br>Instituto de Cardiologia<br>Calle 163 A No. 13 B 60<br>Bogota, Cundinamarca 0<br>COLOMBIA | Comite de Etica en Investigacion Clinica - Fundacion Cardio Infantil<br>Instituto de Cardiologia<br>Departmento de Investigaciones<br>Calle 163 A No. 28-60 Edificio Centro de Investigaciones Piso 5<br>Bogota, Cundinamarca<br>COLOMBIA |
| 1057 | Dr. Luis Diaz Medina | | Dr. Rafael Lince Varela<br>Dr. Miguel Ruz Montes | Clinica Cardiovascular<br>Calle 78 B No. 75-21<br>Medellin, Antioquia 0<br>COLOMBIA | Comite de Etica de la Clinica Cardiovascular<br>Calle 78B No. 75-21<br>Medellin, Antioquia 0<br>COLOMBIA |

090177e1806e1c23\Approved\Approved On: 01-Dec-2008 01:38 (GMT)

## Costa Rica

**Coordinating Investigators:**
<None Entered>

| Center | Principal Investigator | Co-Investigator(s) | Sub-Investigator(s) | Address(es) | Institutional Review Board or Ethics Committee Address(es) |
|---|---|---|---|---|---|
| 1062 * | Dr. Abdon Castro-Bermudez | | Dr. Arnaldo Bonilla-Ibarra<br>Dr. Jorge Faerron-Angel<br>Dr. Regina Gutierrez<br>Dr. Rafael Gutierrez-Alvarez<br>Dr. Francisco Rivera-Valdivia<br>Catalina Sanchez-Soto | Centro Radiologico San Bosco<br>Paseo Colon, de la torre Mercedes Benz 150 mt sur calle 24, Av 2 y 4<br>San Jose, San Jose, Costa Rica<br>COSTA RICA<br><br>Clinica Cordis<br>De la Junta de Proteccion Social, 125 mts oeste<br>San Jose, San Jose, Costa Rica<br>COSTA RICA<br><br>Hospital CIMA San Jose<br>Carretera Prospero Fernandez 500 mts oeste del peaje<br>San Jose, San Jose, Costa Rica<br>COSTA RICA<br><br>Private Medical office<br>Costado Oeste Hospital Nacional de Niños<br>San Jose, San Jose, Costa Rica<br>COSTA RICA | Consejo Nacional de Investigacion en Salud, CONIS<br>Ministerio de Salud<br>Calle 16, Av. 6 y 8<br>San Jose, San Jose, Costa Rica<br>COSTA RICA<br><br>UCIMED<br>Comite Etico Cientifico de la Universidad de Ciencias Medicas<br>400 mts Oeste del Ministerio de Agricultura y Ganaderia<br>Sabana Oeste<br>San Jose<br>COSTA RICA |

* Did not randomize subjects

## Guatemala

**Coordinating Investigators:**
<None Entered>

| Center | Principal Investigator | Co-Investigator(s) | Sub-Investigator(s) | Address(es) | Institutional Review Board or Ethics Committee Address(es) |
|---|---|---|---|---|---|
| 1063 | Dr. Guillermo Gaitan | | Annabella de Arroyo<br>Flor de María Garcia de Castellanos<br>Manuel Ramirez<br>Luis Fernando Rios<br>Dr. Benjamin Torun | Unidad de Cirugia Cardiovascular de Guatemala, UNICAR<br>9 avenida 8-00 zona 11<br>Guatemala, Guatemala<br>GUATEMALA | Latin Ethics<br>Boulevard Vista Hermosa 25-19, Zona 15 Edificio Multimédica, Nivel 4<br>Oficina 409<br>Guatemala 01015, Guatemala<br>GUATEMALA |

090177e1806e1c23\Approved\Approved On: 01-Dec-2008 01:38 (GMT)

090177e1806e1c23\Approved\Approved On: 01-Dec-2008 01:38 (GMT)

## Hungary

**Coordinating Investigators:**
<None Entered>

| Center | Principal Investigator | Co-Investigator(s) | Sub-Investigator(s) | Address(es) | Institutional Review Board or Ethics Committee Address(es) |
|---|---|---|---|---|---|
| 1020 | Dr. Marta Katona | | Dr. Barna Babik<br>Dr. Andras Hari Kovacs<br>Dr. Erzsebet Kertesz<br>Dr. Katalin Racz<br>Dr. Gabor Simon<br>Dr. Attila Somfai | Csongrad County Hospital of Thoracic Diseases<br>Alkotmany ut 36.<br>Deszk, 6722<br>HUNGARY<br><br>University of Szeged, AOK Department of Pediatrics<br>Koranyi fasor 14-15<br>Szeged, 6720<br>HUNGARY<br><br>University of Szeged, Albert Szent-Gyorgyi Medical and Pharmaceutical Centre<br>Department of Ophtalmology<br>Koranyi fasor 14-15.<br>Szeged, 6726<br>HUNGARY | Medical Research Council Ethics Committee for Clinical Pharmacology<br>Arany J. u. 6-8<br>Budapest, H-1051<br>HUNGARY |
| 1021 | Dr. Andras Szatmari | | Dr. Laszlo Ablonczy<br>Dr. Laszlo Kornyei<br>Prof. Ildiko Suveges<br>Dr. Kristof Karlocai | Gyorgy Gottsegen National Institute of Cardiology<br>Haller utca 29<br>Budapest, 1096<br>HUNGARY<br><br>I. Clinic of Ophtalmology<br>Semmelweis University<br>Tomo u. 25-29.<br>Budapest, 1083<br>HUNGARY | Medical Research Council Ethics Committee for Clinical Pharmacology<br>Arany J. u. 6-8<br>Budapest, H-1051<br>HUNGARY |

090177e1806e1c23\Approved\Approved On: 01-Dec-2008 01:38 (GMT)

## India

**Coordinating Investigators:**
<None Entered>

| Center | Principal Investigator | Co-Investigator(s) | Sub-Investigator(s) | Address(es) | Institutional Review Board or Ethics Committee Address(es) |
|---|---|---|---|---|---|
| 1028 | Dr. Kutumba Srinivasa Sastry Bhagavatula | | Ms. Surya Prabha VVS Bharatula<br>Dr. Johann Christopher<br>Ms. Kundana Gogulamudi<br>Mr. Madhan Mohan Kannagunti<br>Nageswara R. Koneti<br>Madhukar K. Reddy<br>Basith A. Siddiqui<br>Dr. Meegada Madan K. Reddy | The Institute of Medical Sciences, CARE Hospital<br>Exhibition Road<br>Namapally<br>Hyderabad, Andra Pradesh 500 001<br>INDIA | Institutional Ethics Committee<br>CARE Foundation - CARE Hospital<br>Road No. 1<br>Banjara Hills<br>Hyderabad , Andhra Pradesh<br>INDIA |
| 1029 | Dr. Krishna Raman Kumar | | Dr. Mahesh Kappannayil<br>Mr. Arun C. Nair<br>Anuradha Suresh Rao<br>Dr. Balu Vaidyanathan<br>Sivadasan R. Anil<br>Dr. Bhava Ramalingam Jawahar Kannan<br>Dr. Ananthen Killikulangara Sadanandan<br>Bhaskaran R. Sajeev<br>Prasanna Sasikumar<br>Archana Thayyil Sudhakaran | Amrita Institute of Medical Sciences & Research Centre,<br>Department of Pediatric Cardiology,<br>Amrita Lane,<br>Elamakkara,<br>Kerala, Kochi, 682 026<br>INDIA | Research and Ethics Committee,<br>Amrita Institute of Medical Sciences & Research Centre<br>Amrita Lane<br>Elamakkara, P.O.<br>Kochi, Kerala 682 026<br>INDIA |

090177e1806e1c23\Approved\Approved On: 01-Dec-2008 01:38 (GMT)

# Italy

**Coordinating Investigators:**
<None Entered>

| Center | Principal Investigator | Co-Investigator(s) | Sub-Investigator(s) | Address(es) | Institutional Review Board or Ethics Committee Address(es) |
|---|---|---|---|---|---|
| 1039 | Dr. Nazzareno Galie' | | Dr. Alessandra Manes<br>Dr. Luca Negro<br>Dr. Federica Pelino<br>Dr. Vincenzo Profazio<br>Dr. Serena Romanazzi<br>Dr. Kia Vaziri Farahani | Dipartimento di Discipline Chirurgiche Rianimatorie e dei Trapianti<br>Sezione di Clinica Oculistica 1 - Policlinico Sant'Orsola<br>Via G. Massarenti, 9<br>Bologna, 40138<br>ITALY<br><br>Policlinico Sant'Orsola<br>Istituto delle Malattie dell'Apparato Cardiovascolare<br>Via G Massarenti 9<br>Bologna, 40138<br>ITALY | Comitato Etico dell'azienda ospedaliera di Bologna - Policlinico S.Orsola-Malpighi<br>Via G. Massarenti, 9<br>Bologna, 40138<br>ITALY |

090177e1806e1c23\Approved\Approved On: 01-Dec-2008 01:38 (GMT)

## Japan

**Coordinating Investigators:**
<None Entered>

| Center | Principal Investigator | Co-Investigator(s) | Sub-Investigator(s) | Address(es) | Institutional Review Board or Ethics Committee Address(es) |
|---|---|---|---|---|---|
| 1071 | Prof. Tsutomu Saji | | Naoki Iino<br>Shinichiro Kobayakawa<br>Dr. Hiroyuki Matsuura<br>Dr. Tomotaka Nakayama<br>Hiromitsu Shimada<br>Shinichi Takatsuki<br>Tetsuo Tochikubo<br>Hiroshi Watanabe | Toho University Omori Medical Center<br>6-11-1<br>Omori-nishi<br>Ota-ku<br>Tokyo, 143-8541<br>JAPAN | Toho University Omori Medical Center IRB<br>6-11-1<br>Ohmori-nishi<br>Ohta-ku, Tokyo 143-8541<br>JAPAN |

090177e1806e1c23\Approved\Approved On: 01-Dec-2008 01:38 (GMT)

## Malaysia

**Coordinating Investigators:**
<None Entered>

| Center | Principal Investigator | Co-Investigator(s) | Sub-Investigator(s) | Address(es) | Institutional Review Board or Ethics Committee Address(es) |
|---|---|---|---|---|---|
| 1024 | Dr. Sim Joo Seng | | Dr. Jonathan Siew-Cheong Choon<br>Dr. Fong Hwa Lai<br>Dr. Toong Chow Lee<br>Dr. Ahmad Tajuddin Othman<br>Dr. Kong Chuan Goh | Gleneagles Medical Center<br>Info Kinetics / Clinical Research Center<br>No 1<br>Jalan Pangkor<br>Penang, Penang 10050<br>MALAYSIA<br><br>Penang General Hospital<br>Psychiatric Unit<br>Residency Road<br>Penang, Penang 10900<br>MALAYSIA | Joint Penang Independent Ethics Committee<br>Clinical Research Center, Gleneagles Medical Center<br>No 1<br>Jalan Pangkor<br>Penang, Penang 10050<br>MALAYSIA |

090177e1806e1c23\Approved\Approved On: 01-Dec-2008 01:38 (GMT)

## Mexico

**Coordinating Investigators:**
<None Entered>

| Center | Principal Investigator | Co-Investigator(s) | Sub-Investigator(s) | Address(es) | Institutional Review Board or Ethics Committee Address(es) |
|---|---|---|---|---|---|
| 1038 | Dr. Tomas Pulido | | Juan Calderon<br>Dr. Maria Cristina Castanon<br>MD Carlos Corona<br>Jose Antonio Garcia<br>Dr. Alejandro Hernandez<br>Dr. Maria Luisa Martinez-guerra<br>MD Roberto Mejía<br>Dr. Tania Rueda<br>Dr. Julio Sandoval<br>Dr. Efren Santos<br>Dr. Edgar Bautista<br>Arturo Carrillo<br>nurse Alicia Castañon<br>Jose Gotes<br>Mateo Porres<br>MD Beatriz Soto | Instituto Nacional de Cardiologia<br>"Dr Ignacio Chavez"<br>Juan Badiano 1<br>Col. Seccion 16<br>Tlalpan, Mexico DF 14080<br>MEXICO<br><br>Instituto Nacional de<br>Enfermedades Respiratorias<br>Calzada de Tlalpan No. 4502.<br>Col. Seccion XVI.<br>Del. Tlalpan, Mexico D.F. 14080<br>MEXICO | Comité de Bioética<br>Instituto Nacional de Cardiologia "<br>Dr. Ignacio Chavez"<br>Juan Badiano 1 Col. Seccion 16<br>Tlalpan<br>Mexico, DF 14080<br>MEXICO<br><br>Comité de Investigación<br>Instituto Nacional de Cardiologia<br>"Dr. Ignacio Chavez"<br>Juan Badiano 1 Col. Seccion 16<br>Tlalpan<br>Mexico, DF 14080<br>MEXICO |

090177e1806e1c23\Approved\Approved On: 01-Dec-2008 01:38 (GMT)

## Poland

**Coordinating Investigators:**
<None Entered>

| Center | Principal Investigator | Co-Investigator(s) | Sub-Investigator(s) | Address(es) | Institutional Review Board or Ethics Committee Address(es) |
|---|---|---|---|---|---|
| 1026 | Prof. Wanda Kawalec | | Dr. Rafal Baranowski<br>Dr. Grazyna Brzezinska-Rajszys<br>Dr. Iwona Korzeniowska-Kubacka<br>Dr. Ewa Rydzewska<br>Dr. Malgorzata Seroczynska<br>Dr Malgorzata Tomyn-Drabik<br>Dr. Anna Turska-kmiec<br>Ms. Aneta Zgodka<br>Dr Maria Zubrzycka<br>Dr. Malgorzata Zuk | Instytut Kardiologii<br>Klinika i Zaklad Rehabilitacji Kardiologicznej i Eletrokardiologii Nieinwazyjnej<br>ul. Alpejska 42<br>Warszawa, 04-628<br>POLAND<br><br>Instytut Pomnik Centrum Zdrowia Dziecka, Klinika Kardiologii<br>Al. Dzieci Polskich 20<br>Warszawa, 04-730<br>POLAND | Komisja Bioetyczna przy Instytucie Pomnik Centrum Zdrowia Dziecka<br>Al. Dzieci Polskich 20<br>Warszawa, 04-730<br>POLAND |
| 1027 | Prof. Jacek Bialkowski | | Dr. Pawel Banaszak<br>Dr. Jacek Baranowski<br>Dr. Beata Chodor<br>Dr. Barbara Jarska<br>Dr. Jacek Kusa<br>Dr. Ewa Markowicz-Pawlus<br>Mariola Pulnar<br>Katarzyna Rycaj<br>Dr. Malgorzata Szkutnik<br>Dr. Teresa Zielinska | Slaskie Centrum Chorob Serca<br>Oddzial Kliniczny Wrodzonych Wad Serca i Kardiologii Dzieciecej<br>ul. Szpitalna 2<br>Zabrze, 41-800<br>POLAND | Komisja Bioetyczna Slaskiego Uniwersytetu Medycznego w Katowicach<br>Poniatowskiego 15<br>Katowice, 40-055<br>POLAND |

090177e1806e1c23\Approved\Approved On: 01-Dec-2008 01:38 (GMT)

| Center | Principal Investigator | Co-Investigator(s) | Sub-Investigator(s) | Address(es) | Institutional Review Board or Ethics Committee Address(es) |
|---|---|---|---|---|---|
| 1030 | Dr. Andrzej Rudzinski | | Dr Halina Kaminska-Mroczkowska<br>Dr. Zbigniew Kordon<br>Dr. Wanda Krol-jawien<br>Dr. Agata Lesniak-sobelga<br>Dr. Malgorzata Mazurek<br>Dr. Elzbieta Olczykowska-Siara<br>Dr. Monika Pieculewicz<br>Dr. Beata J. Pietrucha<br>Dr. Maciej Pitak<br>Prof. Piotr Podolec<br>Maria Trojanowska<br>Dr. Piotr Werynski | Klinika Chorob Serca i Naczyn CM UJ<br>Pracownia Rehabilitacyjno-Diagnostyczna<br>Gabinet Spirometryczno-Metaboliczny<br>ul. Pradnicka 80<br>Krakow, 31-202<br>POLAND<br><br>Uniwersytecki Szpital Dzieciecy w Krakowie<br>Oddzial Kardiologiczny<br>ul. Wielicka 265<br>Krakow, 30-663<br>POLAND<br><br>Uniwersytecki Szpital Dzieciecy w Krakowie<br>Poradnia Okulistyczna<br>ul. Wielicka 265<br>Krakow, 30-663<br>POLAND | Komisja Bioetyczna Uniwersytetu Jagiellonskiego<br>ul. Jagiellonska 10<br>Krakow, 31-010<br>POLAND |

* Did not randomize subjects

## Russian Federation

**Coordinating Investigators:**
<None Entered>

| Center | Principal Investigator | Co-Investigator(s) | Sub-Investigator(s) | Address(es) | Institutional Review Board or Ethics Committee Address(es) |
|---|---|---|---|---|---|
| 1016 | Prof. Yuri M. Belozerov | | Dr. Olga Sergeevna Groznova<br>Prof. Irina M. Shestopalova<br>Dr. Petr Petrovich Skripets<br>Prof. Sergey Nikodimovich Strakhov<br>Dr. Konstanin M. Tutelman | Institute of Paediatrics and Paediatric Surgery<br>Taldomskaya ul., 2<br>Moscow, 127412<br>RUSSIAN FEDERATION | Ethics Committee at the Federal Service on Surveillance in Healthcare and Social Development<br>4, str. 1, Slavyanskaya pl.<br>Moscow, 109074<br>RUSSIAN FEDERATION |
| 1025 * | Prof. Sergey V. Gorbachevsky | | Dr. Oxana Aleksandrovna Ageenkova<br>Vladimir Lvovich Kassil<br>Dr. Inna N. Kireeva<br>Dr. Manolis G. Pursanov<br>Dr. Viktoria Viktorovna Sheverdina<br>Dr. Alexander Vladimirovich Suvorov<br>Dr. Valentina Vladimirovna Timoschenko | Bakoulev Scientific Center for Cardiovascular Surgery RAMS<br>Rublevskoye shosse, 135<br>Moscow, 121552<br>RUSSIAN FEDERATION | The Ethics Committee under Federal Agency of Quality Control Medicines<br>Petrovsky Boulvar 8, korp.3<br>Moscow, 127051<br>RUSSIAN FEDERATION |
| 1051 * | Dr. Galina Igorevna Obraztsova | | Dr. Aelita Berezina<br>Dr. Svetlana Ivanovna Didour<br>Dr. Elena Serogodskaya<br>Dr. Andrey Leonidovich Tsitko | St. Petersburg State Medical Institution<br>Children's Polyclinic #35<br>168/2 Leninskiy Prospec<br>St. Petersburg, 196191<br>RUSSIAN FEDERATION | The Ethics Commitee Under Federal Agency Of Quality Control Medicines<br>Petrovskiy Boulevar, 8 kor.3<br>Moscow, 127051<br>RUSSIAN FEDERATION |



090177e1806e1c23\Approved\Approved On: 01-Dec-2008 01:38 (GMT)

090177e1806e1c23\Approved\Approved On: 01-Dec-2008 01:38 (GMT)



## Sweden

**Coordinating Investigators:**
<None Entered>

| Center | Principal Investigator | Co-Investigator(s) | Sub-Investigator(s) | Address(es) | Institutional Review Board or Ethics Committee Address(es) |
|---|---|---|---|---|---|
| 1019 | Prof. Erkki Pesonen | | Dr. Milad El-Segaier<br>Dr. Olle Pahlm<br>Dr. Kristina Tornqvist | Universitetssjukhuset<br>Barn- och Ungdomscentrum<br>Lund, 221 85<br>SWEDEN | Regionala etikprovningsnamnden i Lund<br>Box 133<br>Lund, 22100<br>SWEDEN |

## Taiwan

**Coordinating Investigators:**
<None Entered>

| Center | Principal Investigator | Co-Investigator(s) | Sub-Investigator(s) | Address(es) | Institutional Review Board or Ethics Committee Address(es) |
|---|---|---|---|---|---|
| 1067 | Prof. Betau Hwang | | Dr. Chien-Chang Juan | Taipei Veterans General Hospital<br>No. 201<br>Section 2, Shih-Pai Road<br>Taipei, Taiwan 11217<br>TAIWAN | Joint Institutional Review Board<br>201 Shih-Pai Road, Section 2<br>Taipei, 112<br>TAIWAN |
| 1068 | Prof. Kai-Sheng Hsieh | | Dr. Wen-Hsien Lu | Kaohsiung Veterans General Hospital<br>No. 386<br>Ta-Chung First Road<br>Kaohsiung, Taiwan 81346<br>TAIWAN | Joint Institutional Review Board<br>201 Shih-Pai Road, Section 2<br>Taipei, 112<br>TAIWAN |
| 1069 | Prof. Mei-Hwan Wu | | Dr. Hsin-Hui Chiu<br>Prof. Jou-Kou Wang | National Taiwan University Hospital, Pediatric Cardiology Division<br>No. 7<br>Chung San South Road<br>Taipei, 100<br>TAIWAN | National Taiwan University Hospital, Ethics Committee<br>7 Chung Shan South Road<br>Taipei, 10012<br>TAIWAN |

090177e1806e1c23\Approved\Approved On: 01-Dec-2008 01:38 (GMT)

## United States

**Coordinating Investigators:**
<None Entered>

| Center | Principal Investigator | Co-Investigator(s) | Sub-Investigator(s) | Address(es) | Institutional Review Board or Ethics Committee Address(es) |
|---|---|---|---|---|---|
| 1001 | Dr. Robyn Joan Barst | | Dr. Erika Suzanne Berman Rosenzweig<br>Dr. Diane Kerstein<br>Dr. Martin Louis Leib<br>Allison C. Widlitz | New York Presbyterian Hospital<br>Columbia University<br>BH-2N<br>3959 Broadway<br>New York, NY 10032<br>UNITED STATES | Western Institutional Review Board<br>3535 Seventh Avenue Southwest<br>Olympia, WA 98502<br>UNITED STATES |
| 1002 * | Dr. Stuart Berger | | Dr. Andrew Nicholas Pelech<br>Dr. Steven David Zangwill | Children's Hospital of Wisconsin<br>MS 713<br>9000 West Wisconsin Avenue<br>Milwaukee, WI 53226<br>UNITED STATES | Children's Hospital of Wisconsin<br>9000 West Wisconsin Avenue<br>Milwaukee, WI 53226<br>UNITED STATES |
| 1003 | Dr. Curtis John Daniels | | Dr. Don Louis Bremer<br>Dr. Stephen Christopher Cook<br>Dr. Timothy Francis Feltes<br>Dr. Richard Philip Golden<br>Dr. Timothy Michael Hoffman<br>Dr. Roozbeh Taeed | Children's Hospital<br>c/o Pediatric Clinical Trials International<br>700 Children's Drive<br>Columbus, OH 43205<br>UNITED STATES<br><br>Clinical Study Center<br>Children's Hospital<br>Suite 6E<br>555 South 18th Street<br>Columbus, OH 43205<br>UNITED STATES | Children's Research Institute<br>Human Subjects Research Committee/CHRF Administration<br>700 Children's Drive<br>Columbus, OH 43205<br>UNITED STATES |

090177e1806e1c23\Approved\Approved On: 01-Dec-2008 01:38 (GMT)

* Did not randomize subjects

090177e1806e1c23\Approved\Approved On: 01-Dec-2008 01:38 (GMT)

| Center | Principal Investigator | Co-Investigator(s) | Sub-Investigator(s) | Address(es) | Institutional Review Board or Ethics Committee Address(es) |
|---|---|---|---|---|---|
| 1004 | Dr. Jeffrey Allan Feinstein | | Dr. Deborah Alcorn<br>Dr. Ramona Loret Doyle<br>Michelle T. Ogawa<br>Dr. John L. Faul<br>Julie T. Roedell | Lucile Packard Children's Hospital Stanford<br>Suite 305<br>750 Welch Road<br>Palo Alto, CA 94304<br>UNITED STATES<br><br>Lucile Packard Children's Hospital at Stanford<br>725 Welch Road<br>Palo Alto, CA 94304<br>UNITED STATES<br><br>Stanford Hospital<br>300 Pasteur Drive<br>Stanford, CA 94305<br>UNITED STATES | Stanford University Medical Center Institutional Review Board<br>1215 Welch Road, MODA<br>Stanford, CA 94305-5401<br>UNITED STATES |
| 1005 | Dr. David Dunbar Ivy | | Dr. J. Brownyn Bateman<br>Dr. Arlene V. Drack<br>Dr. Rebecca Sands-Braverman | The Children's Hospital<br>13123 East 16th Avenue<br>Aurora, CO 80045<br>UNITED STATES | Colorado Multiple Institutional Review Board<br>Building 500, Room N3214<br>13001 E. 17th Place<br>P.O. Box 6508<br>Aurora, CO 80045<br>UNITED STATES |
| 1008 | Dr. David Lloyd Wessel | | Margarita Burmester<br>Dr. Mary Patricia Mullen<br>Ms. Kristi Thomas | Children's Hospital<br>300 Longwood Avenue<br>Boston, MA 02115<br>UNITED STATES | Children's Hospital Boston<br>Committee on Clinical Investigators<br>4th Floor<br>333 Longwood Avenue<br>Boston, MA 02115<br>UNITED STATES |
| 1011 | Dr. Nikoleta Sotirios Kolovos | | Dr. David Thomas Balzer<br>Dr. Paul Checchia<br>Dr. Lawrence Tychsen<br>Carla Driver | Washington University School of Medicine<br>St. Louis Children's Hospital<br>1 Children's Place<br>St. Louis, MO 63110<br>UNITED STATES | Washington University Medical Center IRB<br>Human Studies Committee<br>660 S. Euclid Avenue<br>Box 8089<br>St. Louis, MO 63110<br>UNITED STATES |

* Did not randomize subjects

| Center | Principal Investigator | Co-Investigator(s) | Sub-Investigator(s) | Address(es) | Institutional Review Board or Ethics Committee Address(es) |
|---|---|---|---|---|---|
| 1012 | Dr. Robert Gajarski<br>Dr. Thomas Jonathan Kulik (Previous PI) | | Dr. Steven M. Archer<br>Dr. John Robert Charpie<br>Meg Zamberlan | University of Michigan<br>C.S. Mott Children's Hospital<br>1500 East Medical Center Drive<br>Ann Arbor, MI 48109<br>UNITED STATES<br><br>University of Michigan<br>WK Kellogg Eye Center<br>1000 Wall Street<br>Ann Arbor, MI 48109<br>UNITED STATES | University of Michigan IRB - Medicine<br>University of Michigan Hospitals and Health Systems<br>Argus 1<br>517 West William Street<br>Ann Arbor, MI 48109<br>UNITED STATES |
| 1017 | Dr. Michael Allan Portman MD | | Collette M. Fearneyhough<br>Dr. Troy Alan Johnston<br>Ms. Mary E. Schlater | Children's Hospital Medical Center<br>Cardiology<br>4G-1<br>4800 Sand Point Way NE<br>PO Box 5371/CH11<br>Seattle, WA 98105<br>UNITED STATES | Children's Hospital Medical Center IRB<br>7G-3<br>4800 Sand Point Way NE<br>Seattle, WA 98105<br>UNITED STATES |
| 1040 | Dr. Andrew Martin Atz | | Dr. Jeremy M. Ringewald<br>Dr. Richard L. Saunders<br>Dr. Mae Millicent Winfrey Peterseim<br>Dr. Mark Scheurer | Medical University of South Carolina<br>Children's Hospital<br>Room 601<br>165 Ashley Avenue<br>Charleston, SC 29425<br>UNITED STATES<br><br>Medical University of South Carolina<br>Stormy Eye Center<br>167 Ashley Avenue<br>Charleston, SC 29425<br>UNITED STATES | Medical University of South Carolina<br>Office of Research Integrity<br>Room 501<br>165 Cannon Street<br>Charleston, SC 29425<br>UNITED STATES |



090177e1806e1c23\Approved\Approved On: 01-Dec-2008 01:38 (GMT)

| Center | Principal Investigator | Co-Investigator(s) | Sub-Investigator(s) | Address(es) | Institutional Review Board or Ethics Committee Address(es) |
|---|---|---|---|---|---|
| 1046 * | Dr. J. Donald Moore | | Dr. Thomas P. Doyle<br>Dr. David Gay Morrison | Vanderbilt Children's Hospital<br>Division of Cardiology<br>Suite 5320<br>2200 Children's Way<br>Nashville, TN 37232<br>UNITED STATES | Vanderbilt University Institutional Review Board<br>D-3232 MCN<br>1161 21st Ave. South<br>Nashville, TN 37232-2598<br>UNITED STATES |

090177e1806e1c23\Approved\Approved On: 01-Dec-2008 01:38 (GMT)

* Did not randomize subjects
